# Supplementary material for: Novel Mycoviruses Discovered from a Metatranscriptomics Survey of the Phytopathogenic Alternaria Fungus
Source: Viruses. 2022 Nov 18;14(11):2552. doi: 10.3390/v14112552 (PMC9693364; doi:10.3390/v14112552)
Supplement: Supplementary file 1 [file viruses-14-02552-s001.zip › viruses-2016191-Supplementary Tables/Supplementary Table S1.pdf]

Supplementary Table S1. Origin of the strains of *Alternaria* species used in this study

**Strain**	**Species**	**Origin**	**Host**	**Diseased sample**
AH-14	*Alternaria alternata*	Hefei, Anhui	*Pyrus ussuriensis* × *P. bretschneideri* cv. Chili	leaf
AH-37	*A. alternata*	Hefei, Anhui	*P. ussuriensis* × *P. bretschneideri* cv. Chili	leaf
CQ-34	*A. alternata*	Chongqing	*P. pyrifolia* cv. Xingao	leaf
G-31-2	*A. alternata*	Wuhan, Hubei	*P. pyrifolia* cv. Cuiguan	leaf
GS-23	*A. alternata*	Tianshui, Gansu	*P. ussuriensis* cv. Zaosuli	leaf
GS-24	*A. alternata*	Tianshui, Gansu	*P. ussuriensis* cv. Zaosuli	leaf
HB-13	*A. alternata*	Laohekou, Hubei	*P. pyrifolia* cv. Huanghuali	leaf
HBL-13	*A. alternata*	Laohekou, Hubei	*P. pyrifolia* cv. Huanghuali	leaf
KEL-9-7	*A. alternata*	Akesu, Xinjiang	*P. sinkiangensis*	leaf
SC-32	*A. alternata*	Chengdu, Sichuan	*P. pyrifolia* cv. Jinhua	leaf
SD-4	*A. alternata*	Taian, Shandong	*P. pyrifolia* cv. Huanghuali	leaf
YN-13	*A. alternata*	Dali, Yunnan	*P. pyrifolia* cv. Zaobaimi	flower
YN-3	*A. alternata*	Dali, Yunnan	*P. pyrifolia* cv. Baozhuli	flower
KEL-4-4	*A. arborescens*	Akesu, Xinjiang	*P. sinkiangensis*	leaf
KEL-5-1	*A. arborescens*	Akesu, Xinjiang	*P. sinkiangensis*	leaf
SC-2	*A. arborescens*	Chengdu, Sichuan	*P. pyrifolia* cv. Hohsui	leaf
CQ-33	*A. gaisen*	Chongqing	*P. pyrifolia* cv. Xingao	leaf
AH-20	*A. gaisen*	Hefei, Anhui	*P. ussuriensis* × *P. bretschneideri* cv. Chili	leaf
SC-16	*A. gossypina*	Chengdu, Sichuan	*P. pyrifolia* cv. Jinhua	leaf
SC-10	*A. longipes*	Chengdu, Sichuan	*P. pyrifolia* cv. Hohsui	leaf
AH-16	*A. tenuissima*	Hefei, Anhui	*P. ussuriensis* × *P. bretschneideri* cv. Chili	leaf
AH-25	*A. tenuissima*	Hefei, Anhui	*P. ussuriensis* × *P. bretschneideri* cv. Chili	leaf
AH-28	*A. tenuissima*	Hefei, Anhui	*P. ussuriensis* × *P. bretschneideri* cv. Chili	leaf

AH-29	*A. tenuissima*	Hefei, Anhui	*P. ussuriensis* × *P. bretschneideri* cv. Chili	leaf
AH-6	*A. tenuissima*	Hefei, Anhui	*P. ussuriensis* × *P. bretschneideri* cv. Chili	leaf
CQ-1	*A. tenuissima*	Chongqing	*P. pyrifolia* cv. Huanghuali	leaf
CQ-12	*A. tenuissima*	Chongqing	*P. pyrifolia* cv. Cuiguan	leaf
CQ-13	*A. tenuissima*	Chongqing	*P. pyrifolia* cv. Cuiguan	leaf
CQ-18	*A. tenuissima*	Chongqing	*P. pyrifolia* cv. Cuiguan	leaf
CQ-28	*A. tenuissima*	Chongqing	*P. pyrifolia* cv. Cuiguan	leaf
CQ-4	*A. tenuissima*	Chongqing	*P. pyrifolia* cv. Huanghuali	leaf
DSSL-4	*A. tenuissima*	Akesu, Xinjiang	*P. bretschneideri* cv. Dangshansuli	fruit
G-20-2	*A. tenuissima*	Wuhan, Hubei	*P. pyrifolia* cv. Cuiguan	leaf
G-21-1	*A. tenuissima*	Wuhan, Hubei	*P. pyrifolia* cv. Cuiguan	leaf
G-21-2	*A. tenuissima*	Wuhan, Hubei	*P. pyrifolia* cv. Cuiguan	leaf
G-22-1	*A. tenuissima*	Wuhan, Hubei	*P. pyrifolia* cv. Cuiguan	leaf
G-22-2	*A. tenuissima*	Wuhan, Hubei	*P. pyrifolia* cv. Cuiguan	leaf
G-24-2	*A. tenuissima*	Wuhan, Hubei	*P. pyrifolia* cv. Cuiguan	leaf
G-26	*A. tenuissima*	Wuhan, Hubei	*P. pyrifolia* cv. Cuiguan	leaf
G-27	*A. tenuissima*	Wuhan, Hubei	*P. pyrifolia* cv. Cuiguan	leaf
G-29-1	*A. tenuissima*	Wuhan, Hubei	*P. pyrifolia* cv. Cuiguan	leaf
G-29-2	*A. tenuissima*	Wuhan, Hubei	*P. pyrifolia* cv. Cuiguan	leaf
G-35	*A. tenuissima*	Wuhan, Hubei	*P. pyrifolia* cv. Cuiguan	leaf
G-38-2	*A. tenuissima*	Wuhan, Hubei	*P.* pyrifolia cv. Cuiguan	leaf
G-41	*A. tenuissima*	Wuhan, Hubei	*P. pyrifolia* cv. Cuiguan	leaf
G-5	*A. tenuissima*	Wuhan, Hubei	*P. pyrifolia* cv. Cuiguan	leaf
G-9	*A. tenuissima*	Wuhan, Hubei	*P. pyrifolia* cv. Cuiguan	leaf
GS-17	*A. tenuissima*	Wuwei, Gansu	*P. pyrifolia* cv. Huanghuali	fruit

GS-18	*A. tenuissima*	Wuwei, Gansu	*P. pyrifolia* cv. Huanghuali	fruit
GS-2	*A. tenuissima*	Wuwei, Gansu	*P. pyrifolia* cv. Huanghuali	fruit
GS-3	*A. tenuissima*	Wuwei, Gansu	*P. pyrifolia* cv. Huanghuali	fruit
GS-8	*A. tenuissima*	Wuwei, Gansu	*P. pyrifolia* cv. Huanghuali	fruit
GZ-1	*A. tenuissima*	Guiyang, Guizhou	*P. pyrifolia* cv. Yuanhuang	leaf
GZ-2	*A. tenuissima*	Guiyang, Guizhou	*P. pyrifolia* cv. Yuanhuang	leaf
HB-10	*A. tenuissima*	Laohekou, Hubei	*P. pyrifolia* cv. Huali 2	leaf
HB-15	*A. tenuissima*	Laohekou, Hubei	*P. pyrifolia* cv. Huanghuali	leaf
HB-2	*A. tenuissima*	Wuhan, Hubei	P. pyrifolia cv. Cuiguan	leaf
HB-22	*A. tenuissima*	Wuhan, Hubei	P. pyrifolia cv. Cuiguan	leaf
HB-24	*A. tenuissima*	Wuhan, Hubei	*P. ussuriensis* cv. Nanguoli	leaf
HB-43	*A. tenuissima*	Wuhan, Hubei	*P. bretschneideri*	leaf
HB-45	*A. tenuissima*	Wuhan, Hubei	*P. bretschneideri*	leaf
HB-5	*A. tenuissima*	Laohekou, Hubei	*P. pyrifolia* cv. Huali 1	leaf
HB-6	*A. tenuissima*	Laohekou, Hubei	*P. pyrifolia* cv. Huali 1	leaf
HBL-8	*A. tenuissima*	Laohekou, Hubei	*P. pyrifolia* cv. Huali 2	leaf
JL-5	*A. tenuissima*	Yanbian, Jilin	*P. ussuriensis* cv. Pingguoli	leaf
JL-7	*A. tenuissima*	Yanbian, Jilin	P. *ussuriensis* cv. Pingguoli	leaf
KEL-9-2	*A. tenuissima*	Akesu, Xinjiang	P. *sinkiangensis*	leaf
SC-12	*A. tenuissima*	Chengdu, Sichuan	*P. pyrifolia* cv. Hohsui	leaf
SC-15	*A. tenuissima*	Chengdu, Sichuan	*P. pyrifolia* cv. Hohsui	leaf
SC-21	*A. tenuissima*	Chengdu, Sichuan	*P. pyrifolia* cv. Jinhua	leaf
SC-8	*A. tenuissima*	Chengdu, Sichuan	*P. pyrifolia* cv. Hohsui	leaf
SC-9	*A. tenuissima*	Chengdu, Sichuan	*P. pyrifolia* cv. Hohsui	leaf
SD-14	*A. tenuissima*	Tengzhou, Shandong	*P. bretschneideri* cv. Chazili	leaf

SD-15	*A. tenuissima*	Tengzhou, Shandong	*P. bretschneideri* cv. Chazili	leaf
SD-23	*A. tenuissima*	Yantai, Shandong	*P. bretschneideri* cv. Dabali	fruit
SD-6	*A. tenuissima*	Taian, Shandong	*P. pyrifolia* cv. Huanghuali	leaf
SD-7	*A. tenuissima*	Taian, Shandong	*P. pyrifolia* cv. Huanghuali	leaf
YN-22	*A. tenuissima*	Dali, Yunnan	*P. pyrifolia* cv. Baozhuli	flower
