# Supplementary material for: Novel Mycoviruses Discovered from a Metatranscriptomics Survey of the Phytopathogenic Alternaria Fungus
Source: Viruses. 2022 Nov 18;14(11):2552. doi: 10.3390/v14112552 (PMC9693364; doi:10.3390/v14112552)
Supplement: Supplementary file 1 [file viruses-14-02552-s001.zip › viruses-2016191-Supplementary Tables/Supplementary Table S2.pdf]

Supplementary Table S2 RT-PCR primers used for detection viruses in this study

Primer	Sequence (5′-3′)	Name of virus detected	Target fragments (bp)
contig 5F	TCATCAACACACAATTGCACT	AtHV1	559
contig 5R	AGACGAAAACATTACTACGGA		
contig 13074F	CCAAATGGAAAGGCAACGAA	AtMV1	450
contig 13074R	AAAATTTCATCCATCGGCTAC		
contig 5919F	ATAAAGGAGATTCTTACCGAAC	AtNV1	716
contig 5919R	CACAAAGCGCAAACACTGA		
contig 2423F	CTTTTCTCGCTTAATTCATCGT	AtOLV1	498
contig 2423R	ATTCCAATATTCATCCCTCGT		
contig 2672F	TCCCCAAGAGTAAAGATCCAG	AtOLV2	326
contig 2672R	TTTCGTAATGCGTTCAACCC		
contig 4360F	CCGAGTACCTTCAATTCGTCA	AtOLV3	306
contig 4360R	AAGAAGCTCGATCCACTCA		
contig 6218F	CGTTAGCGAGATCCTAAGACA	AtOLV4	718
contig 6218R	CTCGGAAAGAACCTCAACC		
contig 3454F	GAGTTTGTCACCGTTTCCC	AtOLV5	321
contig 3454R	CTTCACCTCTCGCCAAGCA		
contig 19628F	CACTTGCCTGCTGACAGGACTTCT	AtOLV6	347
contig 19628R	TCTTCAATTAAGCGCAACCTGC		
contig 2521F	AATACGGAGAAATCAATTCCAC	AalOLV1	373
contig 2521R	TCACGGGCCAATACCACAA		
contig 5365F	TCTACTCAAAGGGGCACCA	AarOLV1	721
contig 5365R	CTCGAAATCAACGGAGACGAA		
contig 73F	TTCACCAAGTTGTTTCCGAAG	AtDFV1	486
contig 73R	TGGGCAATCAATACTAGCAC		
contig 13903F	ACGGAGATAGCAATGCCAAA	AaRV1	599
contig 13903R	TCTAATTCCCGCAATAGTCGT		
contig 2756F	CTGCGAGCGGACATGGGTTC	AtCV1	428
contig 2756R	ACATCAGCGTTAGCAGCATA		
contig 19265F	GTTTCCTTAAATTCATTGGCAT	AtPV2	427
contig 19265R	CCTTATATGTACCTTCGTCCA		
contig 7155F	ACTCTAAAACTATTCGACCCT	AaPV1	613
contig 7155R	CGTATAATTTCATGCCATCCCT		
contig 200F	TTCAGCACACTAAGATTACGG	AtBRV2	391
contig 200R	CTACATTTTGAGCAGCGATT		
contig 52F	ACCGAAGCTAATTTGAAACGA	AtNSRV2	663
contig 52R	GCATTCTTCGGGTTTCACT		
contig 35F	TAAAAGAGATGAACCCCGAGA	AtNSRV3	498
contig 35R	ATTTATTCACCACGAAAGCAA		
contig 15899F	TCCCAGCTTCCGCTA	AtNSRV4	618
contig 15899R	TGACAGCACTGAAGAACTC		
contig 192F	ATGTTCCGATCTCTTACAACC	AaNSRV1	389
contig 192R	TACTGATGCCTTGAAGTTGC		
