# Supplementary material for: Novel Mycoviruses Discovered from a Metatranscriptomics Survey of the Phytopathogenic Alternaria Fungus
Source: Viruses. 2022 Nov 18;14(11):2552. doi: 10.3390/v14112552 (PMC9693364; doi:10.3390/v14112552)
Supplement: Supplementary file 1 [file viruses-14-02552-s001.zip › viruses-2016191-Supplementary Tables/Supplementary Table S3.pdf]

Supplementary Table S3. RT-PCR primers used for obtaining full-length sequences of the viruses in this study.

Primer	Sequence (5′-3′)	site of sequence (nt)	Target fragments (bp)
Alternaria tenuissima negative-stranded RNA virus 2 (AtNSRV2)			
contig 52-1F	GCACACCAGCGCAAATGACGA	90 to 107	2550
contig 52-1R	ACTCAGCATTCTTTCTTTCGG	2620 to 2639	
contig 52-2F	TTACTACAATAGCACGTTGGA	2519 to 2538	2528
contig 52-2R	ATTTTCCGTGATGTTCTATTG	5027 to 5046	
contig 52-3F	TCAAATTCAATTGCCGTCTCG	4881 to 4900	1164
contig 52-3R	CGCTAGTCCCGCCATATACTG	6025 to 6044	
contig 52-4F	GTTGCAGAAATTATCGGACCA	5840 to 5859	1255
contig 52-4R	TCATACCTCTCACCACTCTCG	7075 to 7094	
contig 52-5F	TTGATAATTGGAGGTTGACAT	6889 to 6908	1964
contig 52-5R	TTTTAATGACGATTTGCTGTG	8833 to 8852	
contig 52-5′raceR	CATGATGGGAGTCTGCTGGTGG	565 to 585	
contig 52-3′raceF	GCTGCCAACAGTAGCAAAGAGA	8475 to 8495	
Alternaria tenuissima deltaflexivirus 1 (AtDFV1)			
contig 73-1F	TATGTATTTGGCGTGTTGACC	192 to 211	1602
contig 73-1R	CTCGCATGGTAGGAAAGCTTG	1774 to 1793	
contig 73-2F	ACAAACTACCGATTCTGCCTT	1687 to 1706	1596
contig 73-2R	CAAACGAGAAAGCCAACCGAT	3263 to 3282	
contig 73-3-1F	CGGTCTATTTACAGAACAACC	3159 to 3178	851bp
contig 73-3-1R	GTTGGCAGTAGGGATAACACC	3989 to 4008	
contig 73-3-2F	TGTCTTAGCAGCCATTCCCAA	3790 to 3809	1136
contig 73-3-2R	AATACATCATCAGTCGGCTCA	4906 to 4925	
contig 73-4F	GCTGGGTATTGGGGGTGCCTT	4708 to 4727	1775
contig 73-4R	CTCCTCGTCTTCATAGCACCC	6463 to 6482	
contig 73-5F	GATTCCGTGGCGTGTCTCAGC	6384 to 6403	1966
contig 73-5R	ACTCGAAACGGACAAGACACA	8329 to 8349	
contig 73-5′race R1	GGAAACGAACAAACATAGCAA	159 to 178	
contig 73-5′race R2	TTGACCTCCAACAGCCACCAG	228 to 247	
contig 73-3′race F1	TTCCTCTTACCCCTCATTCCG	8201 to 8220	
contig 73-3′race F2	TCACTTCAATTCCTATCAAGC	8140 to 8159	
