# Supplementary material for: Novel Mycoviruses Discovered from a Metatranscriptomics Survey of the Phytopathogenic Alternaria Fungus
Source: Viruses. 2022 Nov 18;14(11):2552. doi: 10.3390/v14112552 (PMC9693364; doi:10.3390/v14112552)
Supplement: Supplementary file 1 [file viruses-14-02552-s001.zip › viruses-2016191-Supplementary Tables/Supplementary Table S4.pdf]

Supplementary Table S4 Abbreviations of virus names and viral protein accession numbers used in alignment analysis in this study

Virus	Abbreviation	accession number
Positive-sense single-stranded RNA virus		
Alternaria tentissima deltaflexivirus 1	AtDFV1	ON263576
Alternaria alternata deltaflexivirus 1	AaDFV1	QTZ98076
Agrostis stolonifera deltaflexivirus 1	AgDFV1	QQG34628
Erysiphe necator associated deltaflexivirus 1	EnADFV1	QKN22722
Erysiphe necator associated deltaflexivirus 3	EnADFV3	QKN22684
Erysiphe necator associated deltaflexivirus 4	EnADFV4	QKN22695
Triticum polonicum deltaflexivirus 1	TpDFV1	QQG34637
Sclerotinia sclerotiorum deltaflexivirus 1	SsDFV1	AMD16208
Negative-sense single -stranded RNA virus		
Alternaria tentissima negative-stranded RNA virus 1	AtNSRV1	QDB75013
Soybean leaf-associated negative-stranded RNA virus 1	SlANSRV1	ALM62220
Fusarium graminearum negative-stranded RNA virus 1	FgNSRV1	ATP75709
Alternaria tentissima negative-stranded RNA virus 2	AtNSRV2	OP566533
Soybean leaf-associated negative-stranded RNA virus 2	SlANSRV2	ALM62227
Cryphonectria parasitica sclerotimonavirus 1	CpNSRV1	QMP84020
Sclerotinia sclerotiorum negative-stranded RNA virus 1	SsNSRV1	AHW76811
Sclerotinia sclerotiorum negative-stranded RNA virus 3	SsNSRV3	AJT39503
Botrytis cinerea negative-stranded RNA virus 3	BcNSRV3	QJT73696
Botrytis cinerea negative-stranded RNA virus 4	BcNSRV4	QJT73698
Sclerotinia sclerotiorum negative-stranded RNA virus 7	SsNSRV7	AWY11040
Botrytis cinerea mymonavirus 1	BcMMV1	AXS76906
Penicillium cairnsense negative-stranded RNA virus 1	PcNSRV1	QDB75012
Plasmopara viticola lesion associated mymonavirus 1	PvLAMM1	QHD64779
