# Supplementary material for: Novel Mycoviruses Discovered from a Metatranscriptomics Survey of the Phytopathogenic Alternaria Fungus
Source: Viruses. 2022 Nov 18;14(11):2552. doi: 10.3390/v14112552 (PMC9693364; doi:10.3390/v14112552)
Supplement: Supplementary file 1 [file viruses-14-02552-s001.zip › viruses-2016191-Supplementary Tables/Supplementary Table S5.pdf]

Supplementary Table S5 Best BLASTp matches of P1 of Alternaria tentissima deltaflexivirts 1

Best hit virus	Protein*	Cover (%)	Identify (%)	E-value	accession number
Agrostis stolonifera deltaflexivirus 1	RdRp	99	98.74	0.0	QQG34628
Alternaria alternata deltaflexivirus 1	RdRp	99	98.25	0.0	QTZ98076
Triticum polonicum deltaflexivirus 1	RdRp	93	96.80	0.0	QQG34637
Erysiphe necator associated deltaflexivirus 1	RdRp	99	95.48	0.0	QKN22722
Erysiphe necator associated deltaflexivirus 3	RdRp	96	68.97	0.0	QKN22684
Sichuan deltaflexi-like virus 2	polyprotein	99	54.08	0.0	QYF50206
Erysiphe necator associated deltaflexivirus 4	RdRp	95	78.14	0.0	QKN22695
Soybean leaf-associated mycoflexivirus 1	RdRp	90	75.47	0.0	YP_009508374
Sclerotinia sclerotiorum deltaflexivirus 1	polyprotein	95	77.25	0.0	UOJ41052
Sesame deltaflexivirus 1	RdRp	94	58.38	0.0	QQG34641
Erysiphe necator associated deltaflexivirts 2	RdRp	86	58.45	0.0	QKN22647
Fusarium graminearum deltaflexivirus 1	polyprotein	86	57.62	0.0	YP_009268710
Agave tequilana deltaflexivirus 1	RdRp	93	53.37	0.0	QQG34632
Xinjiang deltaflexi-like virus 1	polyprotein	88	50.09	0.0	QYF50211
Xinjiang sediment deltaflexi-like virus 2	polyprotein	88	50.00	0.0	QYF50214
Sclerotinia sclerotiorum deltaflexivirus 3	polyprotein	82	41.75	0.0	UOJ41056

* RdRp, RNA-dependent RNA polymerase
