# Supplementary material for: Novel Mycoviruses Discovered from a Metatranscriptomics Survey of the Phytopathogenic Alternaria Fungus
Source: Viruses. 2022 Nov 18;14(11):2552. doi: 10.3390/v14112552 (PMC9693364; doi:10.3390/v14112552)
Supplement: Supplementary file 1 [file viruses-14-02552-s001.zip › viruses-2016191-Supplementary Tables/Supplementary Table S6.pdf]

Supplementary Table S6 Best BLASTp matches of P4 of Alternaria tentissima negative-stranded RNA virus 1

Best hit virus	Best hit protein*	Cover (%)	Identify (%)	E-value	Accession number
Soybean leaf-associated negative-stranded RNA virus 2	RdRp	99	55.85	0.0	ALM62227
Cryphonectria parasitica sclerotimonavirus 1	RdRp	99	56.48	0.0	QMP84020
Sclerotinia sclerotiorum negative-stranded RNA virus 9	RdRp	99	55.83	0.0	QUE49142
Sclerotinia sclerotiorum negative-stranded RNA virus 3	RdRp	99	55.38	0.0	YP_009129259
Botrytis cinerea negative-stranded RNA virus 3	RdRp	99	55.58	0.0	QJT73696
Sclerotinia sclerotiorum negative-stranded RNA virus 1	large polymerase	99	55.46	0.0	YP_009094317
Plasmopara viticola lesion associated mononega virus 2	RdRp	98	54.99	0.0	QHD64785
Fusarium graminearum negative-stranded RNA virus 1	RdRp	97	55.04	0.0	ATP75709
Soybean leaf-associated negative-stranded RNA virus 1	RdRp	97	54.94	0.0	ALM62220
Fusarium asiaticum negative-stranded RNA virus 1	RdRp	97	54.48	0.0	UNG44331
Alternaria tenuissima negative-stranded RNA virus 1	RdRp	99	53.83	0.0	QDB75013
Fusarium proliferatum mymonavirus 1	RdRp	99	53.00	0.0	UWK02084
Botrytis cinerea negative-stranded RNA virus 4	RdRp	99	47.62	0.0	QJT73698
Plasmopara viticola lesion associated mymonavirus 1	RdRp	94	36.00	0.0	QHD64779
Penicillium cairnsense negative-stranded RNA virus 1	RdRp	93	34.69	0.0	QDB75012
Sclerotinia sclerotiorum negative-stranded RNA virus 7	RdRp	92	35.68	0.0	AWY11040
Botrytis cinerea mymonavirus 1	RdRp	92	35.63	0.0	AXS76906

* RdRp, RNA-dependent RNA polymerase
